# Risk factors associated with vitamin D deficiency in preterm neonates: a single-center step-wise regression analysis

**DOI:** 10.1186/s12887-023-04088-w

**Published:** 2023-06-26

**Authors:** Zahra Jamali, Fereshteh Ghorbani, Mohammad Shafie’ei, Fatemeh Tolooefar, Elham Maleki

**Affiliations:** 1 Department of Pediatrics, School of Medicine, Kerman University of Medical Sciences, Kerman, Iran; 2 Nursing Research Center, Kerman University of Medical Sciences, Kerman, Iran; 3 Faculty of Medicine, Kerman University of Medical Sciences, Kerman, Iran; 4 Kerman University of Medical Sciences, Kerman, Iran; 5 Endocrinology and Metabolism Research Center, Institute of Basic and Clinical Physiology Science, Kerman University of Medical Sciences, Kerman, Iran

**Keywords:** Vitamin D deficiency, Pregnancy, Premature newborns

## Abstract

**Background:**

Vitamin D deficiency is particularly concerning in pregnant women, leading to various health-related issues in mothers and their babies, especially those born prematurely, including neonatal skeletal and respiratory disorders. In addition, there have been several reports indicating the presence of multiple impactful factors in the development of vitamin D deficiency. Therefore, we aimed to evaluate the vitamin D level in very preterm and moderately preterm newborns and investigate its association with presumed influential factors.

**Methods:**

This cross-sectional descriptive study was performed on 54 mothers and their preterm neonates with gestational ages less than 34 weeks at delivery (i.e., very preterm and moderately preterm). After the serum vitamin D levels were determined from samples obtained in the first 24 h after birth, the babies were divided into two groups based on the presence or absence of deficiency. The relationship between several factors and the neonatal serum vitamin D level was investigated separately and in a linear step-wise regression model.

**Results:**

The differences between the groups regarding maternal age, gestational age, neonate's gender, birth weight, and delivery method with neonatal vitamin D levels were not statistically significant. However, maternal vitamin D levels strongly correlated with neonatal vitamin D levels (*P*-value < 0.001, *r* = 0.636). The regression model also yielded a strong predictive capability (*P*-value < 0.001, Adjusted *R*^2^ = 0.606), with the maternal vitamin D level demonstrating a significant impact.

**Conclusions:**

Low vitamin D levels in pregnant mothers correlate with deficient levels in their preterm neonates. Therefore, as vitamin D deficiency significantly affects both the mother's and newborn's health, it is recommended that healthcare providers provide comprehensive plans for vitamin D supplementation during pregnancy.

## Introduction

The fat-soluble Vitamin D is produced in the skin by ultraviolet radiation from 7-dehydrocholesterol and then turned into its active form, calcitriol 1, 25 (OH) D, in the liver and the kidneys [1, 2]. It is a vital micronutrient in the intestinal absorption of calcium and phosphorus, strengthening the mineral part of bones [1, 2].

Vitamin D deficiency is a prevalent finding, and approximately 20% of pregnant women and 33% of newborns have serum levels below 25 nmol/l, being burdened with increased risks of related health disorders [3]. Moreover, several factors, such as pregnancy, predispose individuals to the problem [4–7]. Furthermore, low Vitamin D levels are reportedly associated with the onset of several chronic autoimmune disorders, including lupus and multiple sclerosis [4–7].

Vitamin D deficiency during pregnancy is reportedly also associated with an increased risk of pregnancy-induced hypertension, gestational diabetes, abortion, premature birth, low birth weight, and neonatal skeletal and respiratory disorders (due to either inadequate sunlight exposure or nutritional deficiency), as the fetal serum levels are entirely dependent on their mothers' 25-hydroxy vitamin D levels [2, 8–12].

However, these findings have been contradictory to date. For instance, one study found no association between vitamin D levels in the umbilical cord blood of preterm infants and the risk of developing respiratory distress syndrome or bronchopulmonary dysplasia, while others indicated that preterm neonates with deficient serum levels had higher rates of developing the mentioned complications [13–15]. Yet, the mentioned heterogeneity, in our view, should not lead to the allowance of inaction because even if the possibility of such complications was slim, recompensating the mother's already low vitamin D levels with a seemingly easily accessed supplement would lead to favorable results in the mother herself [16–18].

Therefore, investigating the prevalence of impactful factors in newborns' vitamin D levels can be of added interest and importance [7, 19–22]. Several more possible factors have been investigated to date, including birth season, maternal age, gestational age, mother's body mass index, past medical history, serum vitamin D levels, lifestyle, delivery route, and perinatal complications such as preeclampsia [23, 24]. Some studies have also reported various degrees of association between either lower maternal serum levels or gestational ages and subsequently lower vitamin D levels to its severity and effects in preterm neonates [7, 19–22].

Considering the importance of vitamin D deficiency, its high prevalence in pregnant mothers, its effect on fetal growth and development, and the importance of promoting the health of mothers and premature infants in our region (with all pregnant individuals given 1000 daily units of vitamin D supplementation since the detection of pregnancy), we aimed to determine the relationship between vitamin D deficiency in very preterm and moderately preterm infants (gestational ages less than 34 weeks and serum vitamin D levels) as they are at the highest risks of developing it [25–27], providing us with potentially influential factors and means to alter them in our favor.

## Methods

### Study design

We carried out this descriptive cross-sectional study from April 2020 to July 2020 in the regional obstetric-neonatal referral center on pregnant individuals expected to deliver preterm and their preterm neonates with gestational ages less than 34 weeks (i.e., very preterm or moderately preterm) [25].

The local institutional review board approved the study's investigation protocol, data collection, and reporting. Furthermore, written informed consent was obtained from the said mothers and their legal guardians, necessitated due to cultural differences. Moreover, the confidentiality of their personal information and the individual case-by-case results were strictly maintained throughout the study.

Moreover, the laboratory technicians obtaining the serum levels were blinded to the babies' gestational age. Those who participated in the selection of patients and the analysis of the data were also blinded to the individual cases' serum vitamin D levels, and the data was then anonymized by the authors independent from the mentioned processes.

### Participants and sample size determination

The population of interest was mothers admitted to our care center during the mentioned timescale expected to deliver preterm and their preterm neonates with gestational ages less than 34 weeks (i.e., very preterm or moderately preterm as classified by the World Health Organization) [25], who were then selected for eligibility evaluation. This process continued until the desired minimum required sample size was achieved, which was calculated to be *54* by the sample size formula for a quantitative outcome below:

$$n= \frac{4\ {Z}^{2}\ {\sigma }\ ^{2}}{{W}^{2}}$$

In the formula, *Z* was the standard Z-score of the desired level of confidence(i.e., 1.96 for the 95% confidence interval and an *α* of 0.05), *σ* was the available serum vitamin D standard deviation from a similar previously published study by Fallahi et al. [7] which (reported 2.37), and *W* was the margin of error which was calculated using the data provided by the meta-analysis by Badfar et al. [7, 28, 29].

### Inclusion and exclusion criteria

Our inclusion criteria comprised neonates with gestational ages less than 34 weeks (via the first-trimester scan results) delivered naturally or by cesarean section. However, neonates with severe asphyxia, physical or chromosomal abnormalities, meningitis, intraventricular hemorrhage, seizures, sepsis and intubation, and mothers with rheumatologic diseases, including rheumatoid arthritis, thyroid, and parathyroid disorders, liver or cholestatic disease, chronic kidney disease, metabolic bone disease, using anti-retroviral medications during pregnancy, and malabsorption were excluded from the study.

### Data collection and outcome measurement

The participants’ demographic and clinical characteristics were collected from their hospital records. These data included:

- Mothers: mother's age and her number of prior pregnancies along with their outcome, the delivery method, history of steroid or vitamin D intake during pregnancy, along with prenatal onset disorders including gestational diabetes and pregnancy-induced hypertension
- Neonates: gestational age, birth weight, and sex

Moreover, maternal and neonatal serum vitamin D levels were obtained via direct blood sampling. Two milliliters of blood were withdrawn from both mothers (non-fasting basis and in the first 24 h after delivery) and their newly born babies (first 24 h after birth in the neonatal intensive care unit) in identical conditions (i.e., stable hemodynamics and vital signs in the normal ranges while also not in any physical or emotional distress).

The sera were immediately separated in the hospital lab, frozen, and sent to a locally approved laboratory in appropriate environmental conditions. Then, the serum 25 hydroxyvitamin D levels were determined using the enzyme-linked immunosorbent assay (ELISA, BioTec ELx 800, USA) method. Although there is no general agreement on the optimal serum level of Vit D, the Institute of Medicine (IOM) has defined serum vitamin D levels above 20 ng/ml as sufficient, while levels below 20 ng/ml are considered deficient [30]. Moreover, we took into account the normal serum vitamin D reference range of our laboratory (> 20 ng/ml as sufficient and < 20 ng/ml as deficient) along with the most recently published research on the matter [31] and conducted the study in accordance with the IOM definitions, considering vitamin D levels above 20 ng/ml as sufficient and those below 20 ng/ml as deficient. Ultimately, we divided the newborns and their mothers into two groups based on their serum vitamin D levels (i.e., sufficient or deficient) and then investigated their differences.

### Statistical analysis

We analyzed the obtained data using the 21^st^ edition of the SPSS software (SPSS Inc., Chicago, IL). Median and Interquartile Ranges were used to describe the variables without normal distribution, while Mean and Standard Deviation were used to describe those with normal distribution. Mann-Whitney U and Spearman tests were used to investigate the statistical significance level of mean differences and correlation coefficient, respectively. Furthermore, Fisher's Exact test was used to evaluate the differences in qualitative variables between the two groups. Then, step-wise regression analysis was used to assess the predictive effect of the variables in the study on neonatal vitamin D levels. In addition, *P*-values < 0.05 were considered statistically significant.

## Results

We found 54 mothers and their babies eligible for our study, ranging from 16 to 41 years (median = 31). Moreover, most infants were boys (61.1%), delivered via cesarean Sect. (74.1%) with a mean gestational age of 33 weeks and a mean birth weight of 1836.7 ± 488.9 g. Furthermore, the mean maternal and neonatal serum vitamin D levels were 28.62 ± 14.08 ng/ml and 45.3 ± 19.7 ng/ml, respectively, with 20.4% of mothers and 5.6% of neonates being vitamin D deficient. Furthermore, all neonates with deficient vitamin D levels (*n* = 3) were born from mothers with vitamin D deficiency (Table 1).

**Table 1:** Demographic, clinical, and laboratory characteristics of included newborns and mothers

**Variable**				**Total (*n* = 54)**	**Vitamin D sufficient newborns (*n* = 51)**	**Vitamin D deficient newborns (*n* = 3)**	***P*-value** **(Intergroup differences)**
				**Mean ± SD / Median [ IQR] / Frequency (%)**	**Mean ± SD / Median [ IQR] / Frequency (%)**	**Mean ± SD / Median [ IQR] / Frequency (%)**	
**Newborns**	Gender	Boy		33 (61.1%)	30	3	0.155
		Girl		21 (38.9%)	21	0	
	Gestational age (weeks)			33 ± 3.4	32.1 ± 2.03	30 ± 2.65	0.16
				33 [31–34]	33 [31–33]	29 [28 – ?]	
	Birth weight (grams)			1836 ± 488.98	1848.7 ± 492.5	1633.3 ± 450.9	0.493
	Delivery Route	Cesarean section		40 (74.1%)	38 (74.5%)	2 (66.6%)	0.763
		Vaginal (NVD)		14 (25.9%)	13 (25.5%)	1 (33.3%)	
	Neonatal Serum Vitamin D level (ng/mL)			45.3 ± 19.7	47.23 ± 18.74	14.0 ± 5.29	< 0.001***
	Vitamin D group	Sufficient (≥ 20 ng/mL)		51 (94.44%)	-	-	-
		Deficient (< 20 ng/mL)		3 (5.56%)	-	-	
**Mothers**	Maternal age (years)			31.2 ± 2.9	29.61 ± 6.51	30 ± 7.0	0.739
				31 [25.5 – 34]	31 [24.5 – 33.5]	33 [22 – ?]	
	Pregnancy-related adversities	Gestational diabetes	Negative	51 (94.44%)	48 (94.12%)	3 (100%)	0.666
			Positive	3 (5.56%)	3 (5.88%)	0 (0%)	
		Pregnancy-induced hypertension	Negative	49 (90.74%)	47 (92.16%)	2 (66.67%)	0.139
			Positive	5 (9.26%)	4 (7.84%)	1 (33.33%)	
		Taking vitamin D during pregnancy	Negative	26 (48.15%)	24 (47.06%)	2 (66.67%)	0.509
			Positive	25 (46.3%)	27 (52.94%)	1 (33.33%)	
	Receiving antenatal corticosteroids		Negative	24 (44.44%)	23 (45.1%)	1 (33.33%)	0.69
			Positive	30 (55.56%)	28 (54.9%)	2 (66.67%)	
	Maternal Serum Vitamin D level (ng/mL)			28.62 ± 14.08	32.29 ± 14.73	9 ± 1	** < 0.01
	Vitamin D group	Sufficient (≥ 20 ng/mL)		43 (79.6%)	43 (84.3%)	0 (0%)	*** < 0.001
		Deficient (< 20 ng/mL)		11 (20.4%)	8 (15.7%)	3 (100%)	

*Abbreviations*: *SD* standard deviation, *IQR* interquartile range

? The value could not be measured as only three newborns with vitamin D deficiency

^*^ *P*-value < 0.05

^**^ P-value < 0.01

^***^ P-value < 0.001

**Fig. 1:**
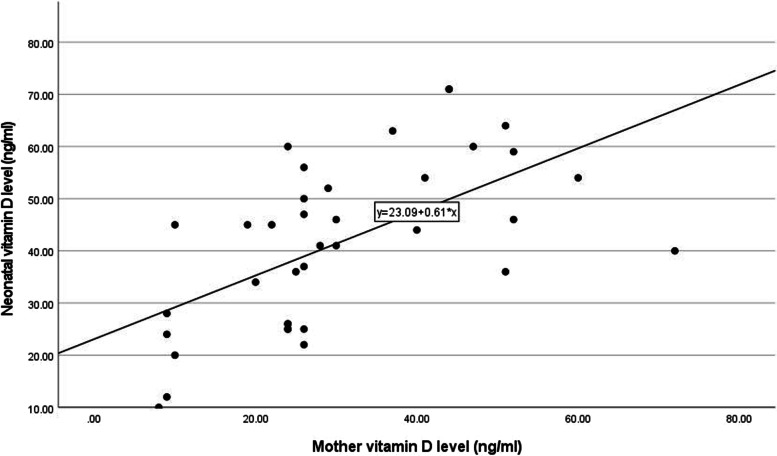
The correlation between neonatal and maternal vitamin D levels

As demonstrated in Tables 1 and 2, the differences between the neonatal vitamin D sufficient and deficient groups regarding maternal age (*P*-value = 0.739), gestational age (*P*-value = 0.16), neonatal gender (*P*-value = 0.155), birth weight (*P*-value = 0.493), or the delivery method (*P*-value = 0.763) were not statistically significant. Moreover, the mean neonatal vitamin D levels were not different based on the newborn's gender (*P*-value = 0.12) or delivery route (*P*-value = 0.99). However, the mean maternal vitamin D level in the deficient group was 9.0 ± 1 ng/ml, while in the sufficient group, the mean level was 32.29 ± 14.73 ng/ml, with the difference between the two groups being statistically significant (*P*-value < 0.01) (Tables 1 and 2) Moreover, we found that maternal vitamin D levels were significantly and positively correlated with the levels in their neonates (*P*-value < 0.001, *r* = 0.636) (Fig. 1).

**Table 2:** Neonatal serum vitamin D levels based on the newborns' sex and delivery method

**Variable**	**Neonatal serum vitamin D level (ng/ml)**	***P*-value**
	Mean ± SD	
**Gender**		
Boy	41.61 ± 18.09	0.12
Girl	51.33 ± 21.27	
**Delivery route**		
Vaginal (NVD)	51.5 ± 23.27	0.99
Cesarean section	43.25 ± 18.25	

*Abbreviations: SD* standard deviation

Finally, only maternal vitamin D levels (*P*-value < 0.001, Adjusted *R*^2^ = 0.606) strongly affected the neonatal serum vitamin D levels in our regression model, with the rest of the variables deemed insignificant and, therefore, omitted in the process.

## Discussion

Vitamin D deficiency in preterm newborns is decisive in many short-term and long-term health-related issues, providing us with the initiative to evaluate potentially influential maternal and prenatal factors.

Compared to similar studies on other populations, we found a considerably smaller proportion of our participants to be vitamin D deficient (approximately 20% of the mothers in our study were deficient, while reports demonstrate a range from 26 to 98%) [32]. Therefore, though the racial, cultural, and regional differences must be considered, the nationwide vitamin D screening and supplementation before and during pregnancy in our region have potentially led to promising results.

Moreover, consistent with previously published studies, we found that not only the maternal serum vitamin D levels are significantly different between vitamin D deficient and sufficient neonates, but it also strongly affects and correlates with the serum level in newly born premature babies [7, 22, 33]. Furthermore, as previously reported by Kotodziejczyk et al. [12], Fallahi et al. [7], and Say et al. [21], premature infants, regardless of their sex, are significantly predisposed to developing vitamin D deficiency, necessitating the undertaking, if not yet so, and the continuation of the policies and measures to mitigate and enhance the negatively and positively impactful factors, respectively.

In summary, our study shows that only the mother's vitamin D level significantly affects that of her neonate, with several studies confirming its effects [7, 33, 34]. However, we did not observe a significant association between cesarean section delivery and neonatal vitamin D level, as reported by Shakeri et al. [9]. These findings suggest that to prevent the previously mentioned health-related issues, all vitamin D deficient pregnant individuals, especially those who are at risk for preterm delivery, should be routinely supplemented with vitamin D. Furthermore, the previously presumed negative correlation of cesarean section deliveries with neonatal serum vitamin D levels should be more comprehensively studied, as even though assuming a linear correlation would be naïve, the presence of other impactful factors on both of the mentioned phenomena is not improbable.

In addition, according to our study, several factors did not affect the vitamin D levels in preterm neonates, namely the mother's age, the gestational age the baby was born with, and the birth weight. Yet, we found these findings controversial as some studies stated results in favor and some against them. El Rifai et al. [33], for example, reported that the gestational age at delivery significantly affects the vitamin level of the neonate, while Anderson-Berry et al. [19] reported otherwise. Shakeri et al. [9] also noted that maternal and neonatal vitamin D levels contrasting with our study, significantly affect the birth weight of the said newborn. These findings, in our view, incentivize further investigation by future studies.

Last, our study also suffered from a few limitations, including its relatively small sample size due to the ongoing pandemic and its single-center design. We believe, therefore, that addressing our deficiencies in future studies would aid more definitive conclusions immensely.

## Conclusions

In cases where premature babies are born, only maternal vitamin D levels significantly correlated with neonatal vitamin D levels among the investigated risk factors of neonatal vitamin D deficiency. Furthermore, due to the decisive effects of vitamin D deficiency on pregnancy outcome and maternal and neonatal health, coherent planning for supplementing mothers with vitamin D deficiency and a suitable follow-up during pregnancy should be highlighted more, for instance, by introducing and strictly implementing comprehensive policies and measures.

## Data Availability

The dataset supporting the conclusions of this article is available upon request to the corresponding author.

## Abbreviations

ELISA: Enzyme-linked immunosorbent assay
AMA: American medical association
